# New gestational diabetes guidelines and misinformed consent

**DOI:** 10.1111/ajo.13236

**Published:** 2020-09-11

**Authors:** Christopher K. Hegerty

**Affiliations:** ^1^ Warwick Hospital Warwick Queensland Australia

I thank Professor Simmons for his interesting response to my article in the June edition of the Journal.^1^ I would like to discuss several points.

Professor Simmons suggests a reduction in perinatal mortality from gestational diabetes mellitus (GDM) treatment. As he says, a non‐significant trend appeared in one trial. However, this hasn’t been reproduced, and none of the deaths in this trial could realistically have been caused by GDM or prevented by treatment, for example a baby with lethal congenital abnormalities.

The best evidence we have for the effect of rising glycaemia on mortality is the HAPO study which Professor Simmons also cites. This, if we are to discuss non‐significant trends, shows no change, or even a decrease in perinatal death as glucose levels rise, as well as a decrease in intrauterine growth restriction. Perinatal mortality may be increased in some women who are also hyperglycaemic, as both can be a consequence of an underlying metabolic syndrome. A superficial assessment may connect the two, but as seems to be the case with other adverse outcomes in GDM women when subjected to multivariable analysis,^2^ hyperglycaemia is likely not a cause but one of the symptoms.

As my article demonstrates, perinatal mortality increases as birthweights decrease. Furthermore, as shown, the practice of pharmacologically restricting the growth of very small babies is common, and it is concerning that some small babies whose development is restrained pharmacologically suffer late stillbirth.^3^

Professor Simmons would tell mothers that GDM diagnosis and treatment unequivocally results in benefit for babies and with no harm.

The Cochrane database disagrees, telling us that the only outcome affecting babies is to reduce the rate of ‘macrosomia’ (with no benefit resulting from this), together with increased labour inductions and with no proven benefit from drug treatment.^4^

Properly informed parents, the great majority with normal‐sized or small babies, may not agree that pharmacologically restricting the development of lean muscle, bone and length in their normal babies is desirable. They may be concerned that some studies suggest this restriction could have long‐term effects on fat distribution and glucose metabolism in their children.

As Professor Simmons says, shoulder dystocia is reduced. However, in the context of GDM this is a technicality, as the decrease of one case for every 48 women treated leads to no benefit for mothers or babies. The same could be said of a reduction in the incidence of hypertension.

As Professor Simmons indicates, the situation regarding caesarean section is unclear. It is possible that even if there were a small effect, some well‐informed and autonomous mothers, rather than have a medicalised pregnancy and three months of drug treatment, may choose to continue with sensible eating and good normal antenatal care and, in the unlikely event that they have a very large baby, choose an elective caesarean section.

As demonstrated in my article, well‐intentioned doctors and midwives, using information provided by government health departments and expert guidelines, are systematically but unwittingly providing misleading non‐evidence‐based information to millions of young women around the world. Unhappily my own state of Queensland is a leading offender.^5^

Professor Simmons cites as scientific justification for the new diagnostic criteria the 2010 expert consensus based on the HAPO study. The criteria used to increase the diagnosis rate did not include mortality, trauma, hypoglycaemia, caesarean section, long‐term health or any other important outcomes, but were instead based on the three clinically meaningless surrogates of ‘birthweight above the 90th percentile, cord C‐peptide and fat percentage’.

The HAPO authors themselves admit that the first two can be viewed as ‘physiological consequences of glycemia rather than as true disorders or problems’, while higher neonatal fat percentage causes no known harm and may be beneficial.^6^

A single Spanish hospital study is mentioned, claiming benefits and reduced costs by increasing the diagnosis rate to 35% of pregnancies. While this may demonstrate economies of scale, other studies, including the recent Australian studies quoted in the article, show the opposite.

Professor Simmons suggests awaiting the outcome of the TOBOGM study before contemplating change. This study, whatever the outcome, involves a different type of woman, diagnosed differently and treated differently, and so is irrelevant to the present discussion. We have the evidence we need.

In conclusion, despite Professor Simmons’ eloquent assertion to the contrary, the important question remains, is a diagnosis and treatment with minimal or no benefit worth the potential for harm?

Funding: none.
